# Comprehensive Analysis of Autophagy-Related Genes in Sweet Orange (*Citrus sinensis*) Highlights Their Roles in Response to Abiotic Stresses

**DOI:** 10.3390/ijms21082699

**Published:** 2020-04-13

**Authors:** Xing-Zheng Fu, Xue Zhou, Yuan-Yuan Xu, Qiu-Ling Hui, Chang-Pin Chun, Li-Li Ling, Liang-Zhi Peng

**Affiliations:** 1 Citrus Research Institute, Southwest University, Chongqing 400712, China; yokie_x@outlook.com (X.Z.); xuyuanyuan19971004@163.com (Y.-Y.X.); 13430231743@163.com (Q.-L.H.); chunchangpin@cric.cn (C.-P.C.); linglili@cric.cn (L.-L.L.); pengliangzhi@cric.cn (L.-Z.P.); 2 Citrus Research Institute, Chinese Academy of Agricultural Sciences, Chongqing 400712, China

**Keywords:** citrus, autophagy, ATG18a, ATG18b, drought, heat, cold, heavy metal

## Abstract

Autophagy is a highly conserved intracellular degradation pathway that breaks down damaged macromolecules and/or organelles. It is involved in plant development and senescence, as well as in biotic and abiotic stresses. However, the autophagy process and related genes are largely unknown in citrus. In this study, we identified 35 autophagy-related genes (*CsATGs—*autophagy-related genes (*ATGs*) of *Citrus sinensis*, Cs) in a genome-wide manner from sweet orange (*Citrus sinensis*). Bioinformatic analysis showed that these CsATGs were highly similar to *Arabidopsis* ATGs in both sequence and phylogeny. All the *CsATGs* were randomly distributed on nine known (28 genes) and one unknown (7 genes) chromosomes. Ten *CsATGs* were predicted to be segmental duplications. Expression patterns suggested that most of the *CsATG* were significantly up- or down-regulated in response to drought; cold; heat; salt; mannitol; and excess manganese, copper, and cadmium stresses. In addition, two *ATG18* members, *CsATG18a* and *CsATG18b*, were cloned from sweet orange and ectopically expressed in *Arabidopsis*. The *CsATG18a* and *CsATG18b* transgenic plants showed enhanced tolerance to osmotic stress, salt, as well as drought (*CsATG18a*) or cold (*CsATG18b*), compared to wild-type plants. These results highlight the essential roles of *CsATG* genes in abiotic stresses.

## 1. Introduction

Autophagy is a highly conserved intracellular degradation process that occurs in eukaryotes including yeasts, animals, and plants [1,2]. During this process, damaged macromolecules and/or organelles are sequestered into a double-membrane vesicle (called an autophagosome) and then delivered into a vacuole (yeast and plants) or a lysosome (animals) for breakdown. The cellular cytoplasmic contents are then recycled [1,2,3,4]. Autophagy-related genes (*ATGs*) are responsible for the regulation and control of the autophagy process. The first *ATG* gene was identified from yeast, and at least 32 *ATGs* have been shown to participate in yeast autophagy [5,6]. To date, many homologues of *ATGs* have been identified from various plant species, including 40 *AtATGs* in *Arabidopsis* [1,3] (*Arabidopsis thaliana*, At), 33 *OsATGs* in rice [7] (*Oryza sativa*, Os), 30 *NtATGs* in tobacco [8] (*Nicotiana tabacum*, Nt), 45 *ZmATGs* in maize [9] (*Zea mays*, Zm), 29 *CaATGs* in pepper [10] (*Capsicum annuum*, Ca), 37 *SiATGs* in foxtail millet [11] (*Setaria italic*, Si), 32 *MaATGs* in banana [12] (*Musa acuminate*, Ma), and 35 *VvATGs* in grapevine [13] (*Vitis vinifera*, Vv). According to the reported characterizations of *ATGs* in yeast and *Arabidopsis*, these *ATGs* can be divided into the following functional groups: (1) the ATG1/13 kinase complex consisting of ATG1, ATG13, ATG20, and TOR (target of rapamycin kinase), mainly functioning on autophagy induction and initiation; (2) the PI3K (phosphatidylinositol 3 kinase) complex consisting of ATG6, VPS15 (vacuolar protein sorting-associated protein), and VPS34 that is involved in vesicle nucleation and autophagosome formation; (3) the ATG9/2/18 complex consisting of ATG9, ATG2, and ATG18 that is responsible for the delivery of membranes for autophagosome formation; (4) the ubiquitin-like ATG8-PE (phosphatidylethanolamine) conjugation pathway (including ATG3, ATG4, ATG7, and ATG8) and ATG12–ATG5 conjugation pathway (including ATG5, ATG7, ATG10, ATG12, and ATG16) that are involved in the elongation of autophagic vesicles; and (5) the VTI12 (vesicle transport v-soluble *N*-ethylmaleimide-sensitive-factor attachment protein receptor (SNARE) protein) family belonging to the SNARE group, which contributes to the fusion of autophagosomes with vacuoles [1,2,3,4,6].

The autophagy process maintains a basal level under normal conditions, but it can be quickly induced by external stimuli such as nutrition starvation, drought, salt, heat, and oxidative and osmotic stresses [3,12,14,15,16]. Many studies have demonstrated the functions of ATGs in alleviating abiotic stresses in plants. For example, an apple *MdATG8i* (*Malus domestica*, Md) was significantly up-regulated in response to nitrogen depletion and oxidative stress, and overexpression of *MdATG8i* in apple callus conferred enhanced tolerance to nutrient-limited conditions [17]. *MdATG3a*, *MdATG3b*, *MdATG7b*, and *MdATG18a* of apple were shown to confer tolerance to nitrogen/carbon starvation, drought, salt, or osmotic stresses by ectopic-expressing in *Arabidopsis* and apple [18,19,20,21]. Li et al. reported that a banana *MaATG8f* improved drought stress tolerance in transgenic *Arabidopsis* through modulating reactive oxygen species (ROS) metabolism, abscisic acid biosynthesis, and autophagy activity [22]. Chen et al. overexpressed an *ATG8* in *Arabidopsis* that increased autophagic activity and improved nitrogen remobilization efficiency [23]. Moreover, overexpression of a *SiATG8a* of foxtail millet conferred tolerance to both nitrogen starvation and drought in transgenic *Arabidopsis* and rice [11,24]. In contrast, silencing of *AtATG18a* in *Arabidopsis* reduced drought and salt tolerance [25]. Knockdown of the *ATG6* of wheat inhibited autophagy and resulted in accelerated programmed cell death (PCD) under drought stress [26]. Loss of *ATG10* and *ATG18f* significantly reduced *HsfA1a*-induced drought tolerance and autophagosome formation in tomato [27]. Under salt stress, autophagosome formation was rapidly induced, and the atg2 and atg7 mutants of *Arabidopsis* showed hypersensitive phenotype [28]. Under heat stress, atg mutants of *Arabidopsis* displayed visibly impaired pollen development (including *atg2-1*, *atg5-1*, a*tg7-2*, and *atg10-1*) [29] or enhanced growth inhibition (including *atg2-1*, *atg5-1*, *atg12ab*, and *atg18a-2*) [30]. Recently, Shinozaki et al. proved that autophagy played important role in maintaining zinc bioavailability to avoid ROS accumulation under zinc deficiency in *Arabidopsis* [31]. Moreover, the potential roles of autophagy in waterlogging and excess aluminum and copper stresses were also reported [13,32,33].

As the largest fruit crop, citrus is widely grown around the world. In 2017, the global citrus growing area and yield were 14.1 million hectares and 146.6 million tons, respectively (FAO statistics, http://faostat.fao.org/default.aspx). However, citrus production is limited by abiotic stresses including drought, heat, cold, and nutrient disorders. Although the underlying physiological and molecular mechanisms triggered in response to these stresses have been well studied, the autophagy process and *ATG* genes in citrus are largely unknown. In this study, we systematically identified 35 *ATGs* from citrus by using the genome of sweet orange [34]. Comprehensive analysis including bioinformatic characterizations and expression profiles of these *ATGs* were conducted and discussed. Moreover, two *ATGs* of sweet orange, *CsATG18a* and *CsATG18b* (*Citrus sinensis*, Cs), were cloned and overexpressed in *Arabidopsis*. This further supported their functions in improving tolerance to drought, salt, cold, and osmotic stresses.

## 2. Results

### 2.1. Identification of 35 ATGs in the Sweet Orange Genome

**Figure 1:**
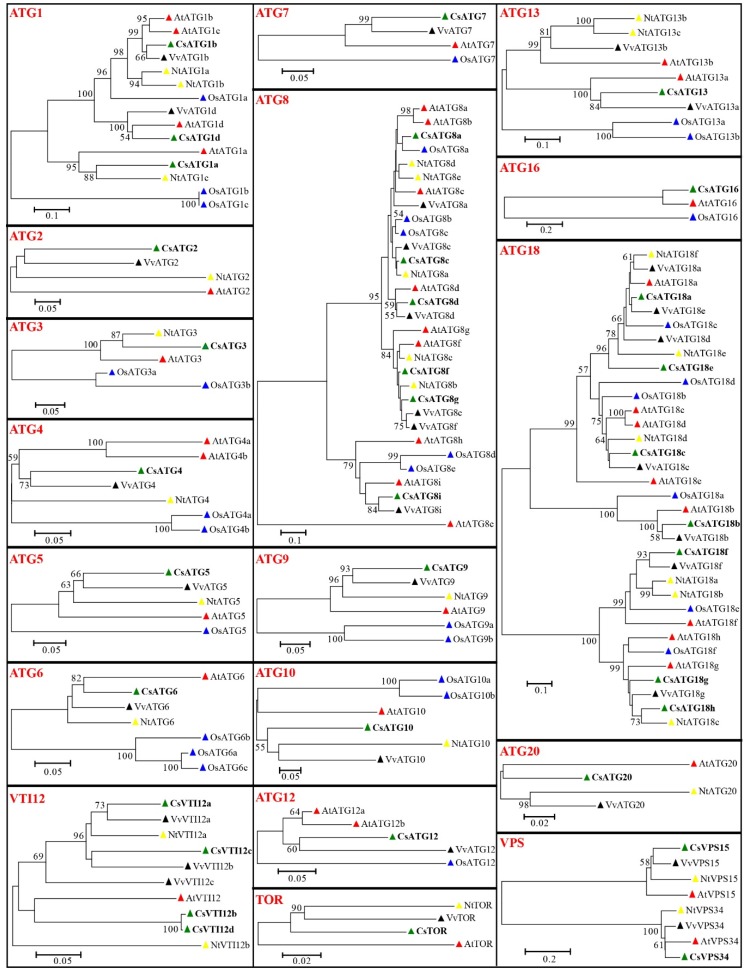
Phylogenetic analysis of sweet orange and other plant ATG proteins. Thirty-five CsATGs of sweet orange (*Citrus sinensis*, Cs), 40 AtATGs of *Arabidopsis thaliana* (At), 30 NtATGs of tobacco (*Nicotiana tabacum*, Nt), 31 OsATGs of rice (*Oryza sativa*, Os), and 33 VvATGs of grapevine (*Vitis vinifera*, Vv) were used to construct the neighbor-joining tree in MEGA 6.0 software, with 1000 bootstrap replicates. The same plant of ATGs are marked with the same color of triangles. The bootstrap values (the number at each node) higher than 50% are shown in the phylogenetic trees.

After a BLASTP search of the sweet orange genome with known AtATG proteins as queries and confirmation of the existence of the ATG domains in the PFAM database, a total of 35 putative ATG proteins were identified from sweet orange (Table 1). These ATGs showed 47.6% to 90.9% sequence identity with AtATGs, and they were named and numbered as CsATGs according to the highest sequence identity and the closest phylogenic relationship of AtATG homologues (Table 1 and Figure 1). On the basis of known functional classification of AtATGs, all CsATGs were also classified in the categories of the ATG1/13 kinase complex, PI3K complex, ATG9/2/18 complex, ubiquitin-like ATG8 and PE conjugation pathway, ubiquitin-like ATG12 and ATG5 conjugation pathway, and SNARE (Table 1). Among the 35 CsATGs, 15 contained only one member, whereas 4 CsATGs were encoded by gene families (three members for CsATG1, six members for CsATG8, seven members for CsATG18, and four members for CsVTI12). The ORF length of the *CsATGs* ranged from 315 bp (*CsATG12*) to 7095 bp (*CsTOR*), encoding 104 to 2364 amino acids (Table 1). This suggested the existence of significant variations and potential function differentiation. Predicted subcellular localization suggested that most CsATGs express in the cytoplasm, but there are also members located in the mitochondria, plasma membrane, Golgi apparatus, vacuoles, and nucleus (Table 1).

### 2.2. Bioinformatic Characterizations of CsATGs

Neighbor-joining phylogenetic trees were constructed with 35 CsATGs, 40 AtATGs, 30 NtATGs, 31 OsATGs, and 33 VvATGs, and the results showed that most of the CsATG proteins closely clustered with their homologues in *Arabidopsis* and grapevine (Figure 1). For the ATGs containing multiple members (such as CsATG1, CsATG8, CsATG18, CsVTI12, and CsVPS in sweet orange), the different members of same subfamily were clustered in two or three different branches. For example, three CsATG1 members were clustered in two branches (CsATG1b and CsATG1d in one branch, and CsATG1a in another branch), and seven CsATG18 members were clustered in three branches (CsATG18a, CsATG18c, and CsATG18e in one branch; CsATG18f, CsATG18g, and CsATG18h in one branch; and CsATG18b in another branch). This result suggests potential functional differentiations in these subfamilies. 

**Figure 2:**
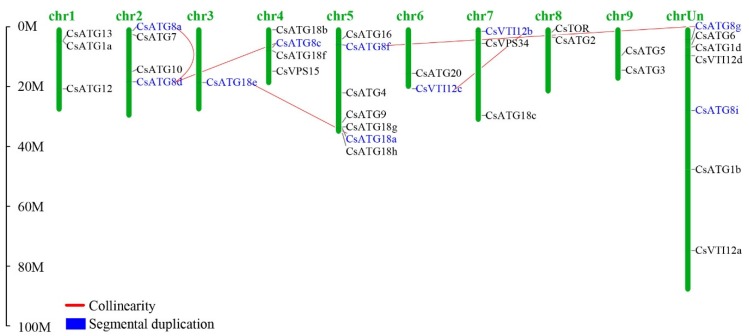
Chromosomal distribution and gene duplication of *CsATG* genes. The genes highlighted with blue color represent segmental duplications. The pairs linked with red lines represent collinear genes.

The chromosomal distribution of 35 *CsATGs* is shown in Figure 2. In total, 28 *CsATGs* were distributed on 9 chromosomes, whereas the chromosomal location of 7 *CsATGs* were unknown. Chromosome 5 (chr5) contained the most *CsATGs* (seven genes), followed by chr2 (four genes) and chr4 (four genes). Gene duplication analysis showed that 10 *CsATGs* were predicted to be segmental duplicated genes, accounting for about 29% of all *CsATGs*. In these segmental duplicated genes, five pairs—*CsATG8a* and *CsATG8d*, *CsATG8c* and *CsATG8d*, *CsATG18a* and *CsATG8e*, *CsATG8f* and *CsATG8g*, and *CsVTI12b* and *CsVTI12c*—were predicted to have collinear correlations.

**Figure 3:**
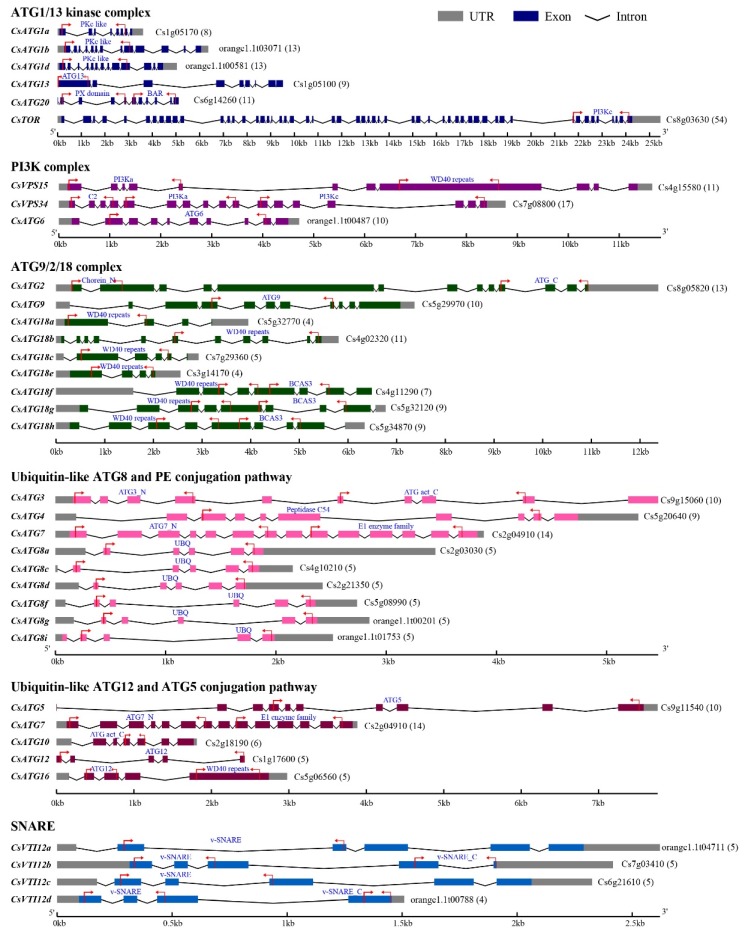
Gene structures and conserved domains of CsATGs. The detailed intron-exon structures and domains of all CsATGs were predicted in the Gene Structure Display Server and the NCBI Conserved Domain Database, respectively. The results are shown and listed according to their functional classifications. Arabic numerals in the parentheses after each gene ID represent exon number. The arrows show the start and end positions of domains. Full names of the conserved domains are listed in the Abbreviations section.

The detailed intron-exon characterizations and conserved domains of CsATGs are shown in Figure 3. We found that 34 of the *CsATGs* had 4 to 17 exons, but 1 gene (*CsTOR*) had 54 exons. All the *CsATG* genes contained introns, but three members (*CsATG12*, *CsATG13*, and *CsATG20*) had no untranslated region (UTR). The main conserved domains of CsATGs were predicted to be protein kinase superfamily (PKc)-like, ATG, phox homology (PX) domain, Bin/amphiphysin/Rvs (BAR), PI3K, WD40, breast carcinoma amplified sequence 3 (BCAS3), chorein N, peptidase C54, ubiquitin homologues (UBQ), E1 enzyme family, and vesicle transport v-SNARE (V-SNARE). In general, the CsATGs in the same functional group or subfamily had similar domains (Figure 3).

### 2.3. Expression Patterns of CsATGs Under Abiotic Stresses

**Figure 4:**
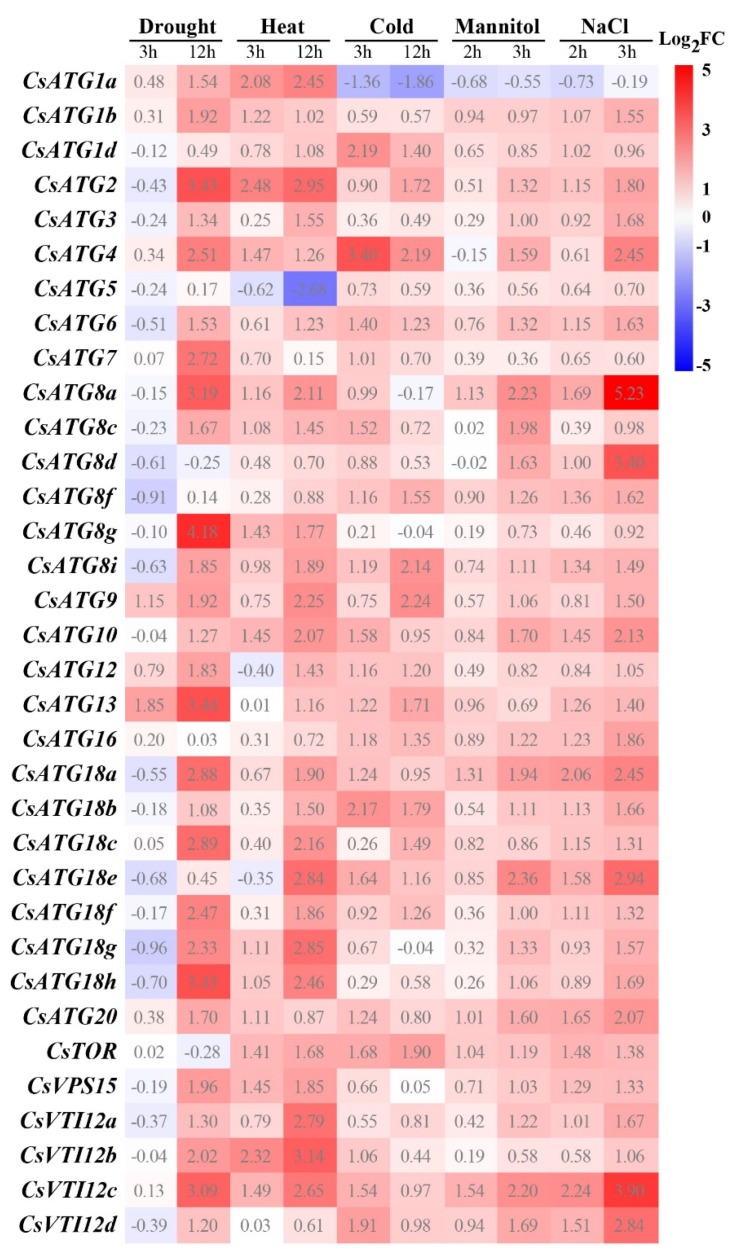
Expression patterns of *CsATG* genes under drought, heat, cold, mannitol, and salt stresses. qRT-PCR was used to determine the relative expression levels of *CsATGs* in the leaves of sweet orange treated with drought, heat (42 °C), cold (4 °C), mannitol (400 mM), and NaCl (200 mM) stresses. Leaves sampled from plants under normal conditions were used as controls (CK). The fold change (FC) was calculated as (relative expression of *CsATGs* under treatments)/(relative expression of *CsATGs* in the CK). The R package was used to generate the heat map based on the mean log_2_FC values of three biological replicates.

As shown in Figure 4, most of *CsATG* genes were up-regulated under different stresses. For example, 27, 28, 23, 22, and 29 of *CsATGs* were significantly up-regulated (log_2_FC > 1) during at least one time-point of drought, heat, cold, mannitol, or salt treatments, respectively. In particular, *CsATG2*, *CsATG8g*, *CsATG13*, and *CsATG18h* under drought; *CsATG2*, *CsATG18e*, *CsATG18g*, and *CsVTI12b* under heat; *CsATG4* under cold; and *CsATG8a*, *CsATG18e*, and *CsVTI12*c under mannitol and salt showed high expression levels, suggesting their potential key roles in response to these stresses. Apart from *CsATG1d*, *CsATG5*, *CsATG7*, *CsATG8d*, *CsATG8g*, *CsATG16*, *CsATG18e*, *CsTOR*, and *CsVTI12d*, the other 26 tested genes were significantly up-regulated during both drought and heat stresses. Twenty *CsATGs* showed significant up-regulation under both mannitol and salt stress. Remarkably, *CsATG2*, *CsATG4*, *CsATG8i*, *CsATG9*, *CsATG10*, *CsATG18a*, *CsATG18b*, and *CsVTI12c* were significantly up-regulated under all tested stresses simultaneously, suggesting their important roles in response to these stresses. In addition, some genes were significantly up-regulated at all tested time points of treatment, such as *CsATG9* and *CsATG13* under drought. Apart from these up-regulated *CsATGs*, we also noticed that *CsATG1a* was down-regulated under cold, mannitol, and salt treatments, which differed from the expression of the other two members of the ATG1 subfamily, *CsATG1b* and *CsATG1d*. The expression of *CsATG5* was not obvious (absolute value of log_2_FC < 1) under most of the stresses, except for a significant down-regulation under 12 h of heat treatment. Similarly, the expression of *CsATG8d* was also steady, except for a significant up-regulation under mannitol and salt treatments.

**Figure 5:**
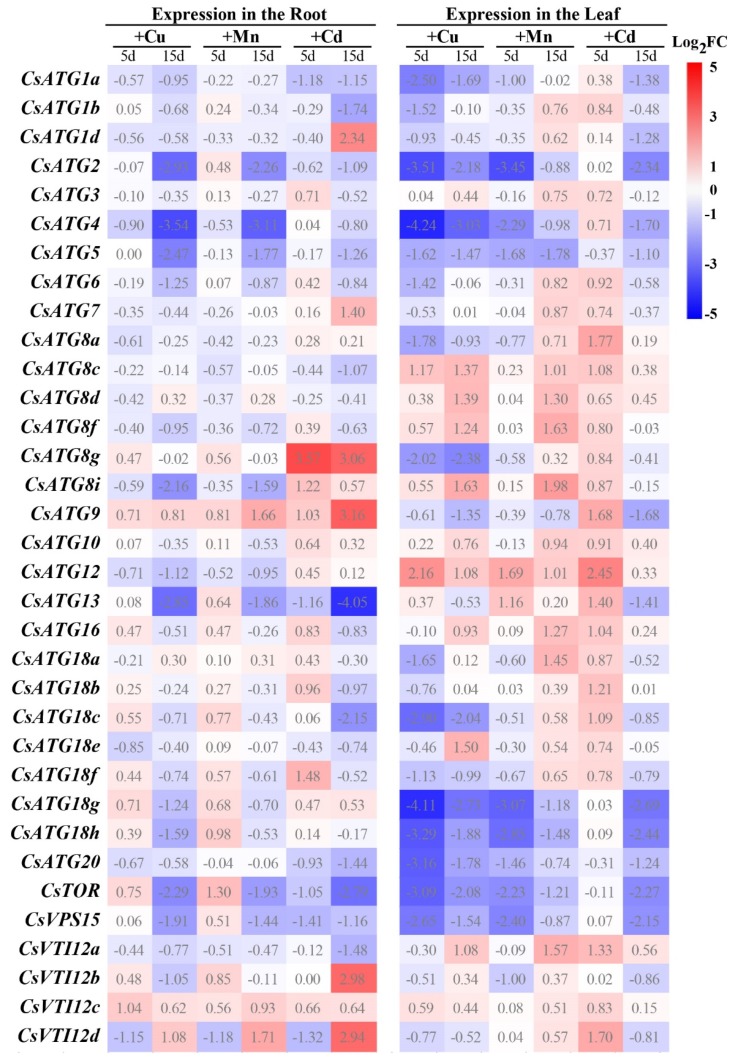
Expression patterns of *CsATG* genes under excess Cu, Mn, and Cd stresses. qRT-PCR was used to determine the relative expression levels of *CsATGs* in the roots and leaves of sweet orange treated with excess Cu, Mn, and Cd for 5 and 15 days. The roots or leaves sampled from plants under normal conditions for 5 and 15 days were used as controls (CK). The fold change (FC) was calculated as (relative expression of *CsATGs* under treatments)/(relative expression of *CsATGs* in the CK). The R package was used to generate a heat map based on the mean log_2_FC values of three biological replicates.

Under excess Cu (+Cu), Mn (+Mn), and Cd (+Cd) treatments, the expression of most *CsATGs* were down-regulated or not obvious in roots and leaves, and only a few *CsATGs* were significantly up-regulated at one or two time-points of treatment (Figure 5). Generally, *CsATG2*, *CsATG4*, *CsATG5*, *CsTOR*, and *CsVPS15* were significantly down-regulated (log_2_FC < -1) in both roots and leaves, *CsATG18g*, *CsATG18h*, and *CsATG20* were significantly down-regulated in leaves, and *CsATG13* was significantly down-regulated in roots under +Cu, +Mn, and +Cd stresses simultaneously. In addition, the expression of *CsATG1a* in +Cd roots and +Cu leaves, as well as *CsATG8g* and *CsATG18c* in +Cu leaves showed extreme down-regulation. Expression of *CsATG8g* and *CsATG9* in +Cd roots, *CsATG8c* in +Cu leaves, and *CsATG12* in both +Cu and +Mn leaves showed obvious up-regulation (Figure 5). These genes may play roles in response to heavy metal stress.

### 2.4. Overexpression of CsATG18a/b Conferred Osmotic and Salt Tolerance in Arabidopsis

**Figure 6:**
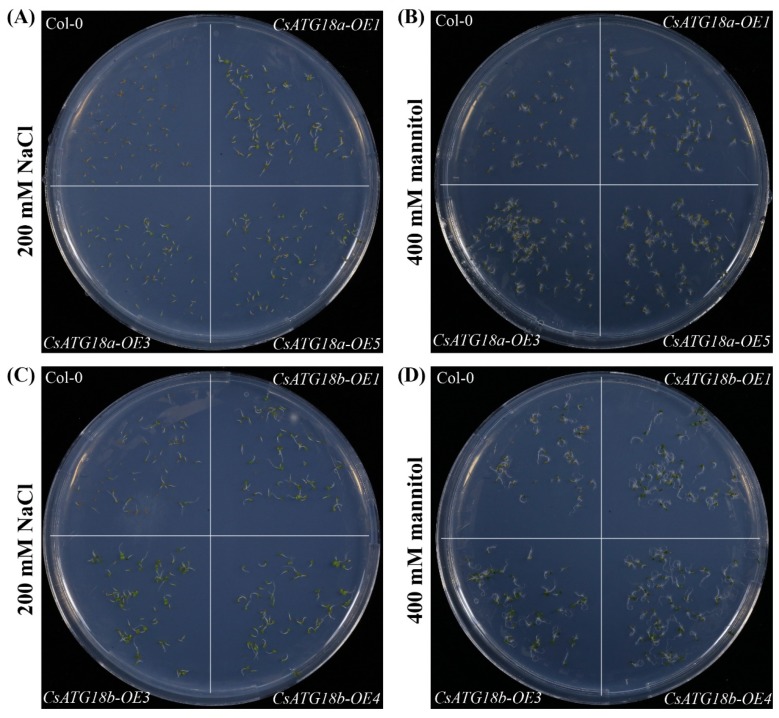
Germination phenotype of *CsATG18a*- and *CsATG18b*-overexpressed (OE) lines and wild-type (WT) under NaCl and mannitol stresses. (**A–D**) Representative pictures of three homozygous OE lines (OE1, OE3, and OE5 of *CsATG18a*, and OE1, OE3, and OE4 of *CsATG18b*) and WT (Col-0) of *Arabidopsis* that germinated on 200 mM NaCl (**A,C**) and 400 mM mannitol (**B,D**) for 7 days. Three independent experiments were performed and the similar phenotype was observed.

**Figure 7:**
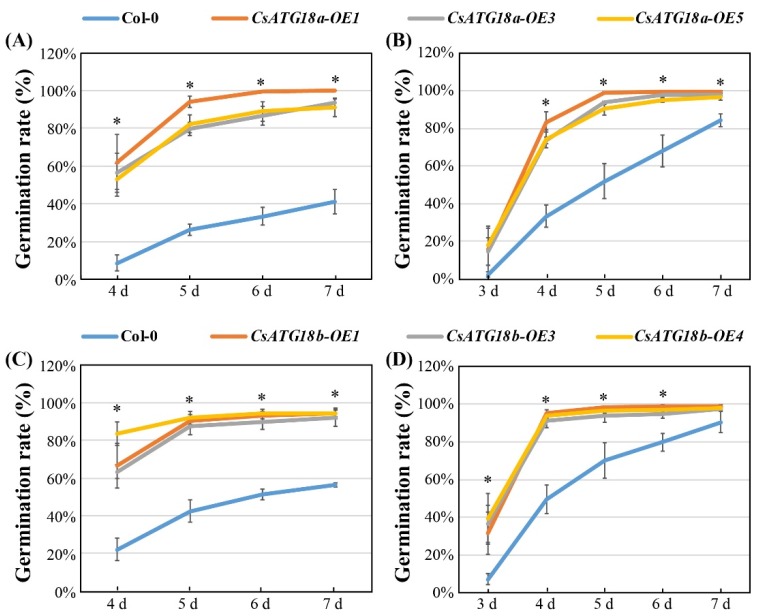
Germination rate of *CsATG18a*- and *CsATG18b*-overexpressed (OE) lines and WT under NaCl and mannitol stresses. (**A–D**) Germination rate (%) of three OE lines of *CsATG18a* and *CsATG18b* and WT (Col-0) calculated after 4, 5, 6, and 7 days of 200 mM NaCl (**A,C**) treatment and 3, 4, 5, 6, and 7 days of 400 mM mannitol (**B,D**) treatment. The data are the means ± SE of three independent repeats (*n* = 60). * indicates a significant difference at *p* < 0.05 using the least significant difference (LSD) test.

To confirm the functions of *CsATGs* in abiotic stresses, *CsATG18a* and *CsATG18b* were cloned and overexpressed in *Arabidopsis*. Three homozygous overexpression (OE) lines (T4 generation) and wild-type (WT, Col-0) of *Arabidopsis* were selected to perform germination experiments. The results showed that the OE lines of *CsATG18a* (OE1, OE3, and OE5) and *CsATG18b* (OE1, OE3, and OE4) had significantly higher germination rates than WT on 200 mM NaCl and 400 mM mannitol (Figure 6 and Figure 7). These data suggest that overexpression of both *CsATG18a* and *CsATG18b* confer osmotic and salt tolerance.

### 2.5. Overexpression of CsATG18a Conferred Drought Tolerance in Arabidopsis

**Figure 8:**
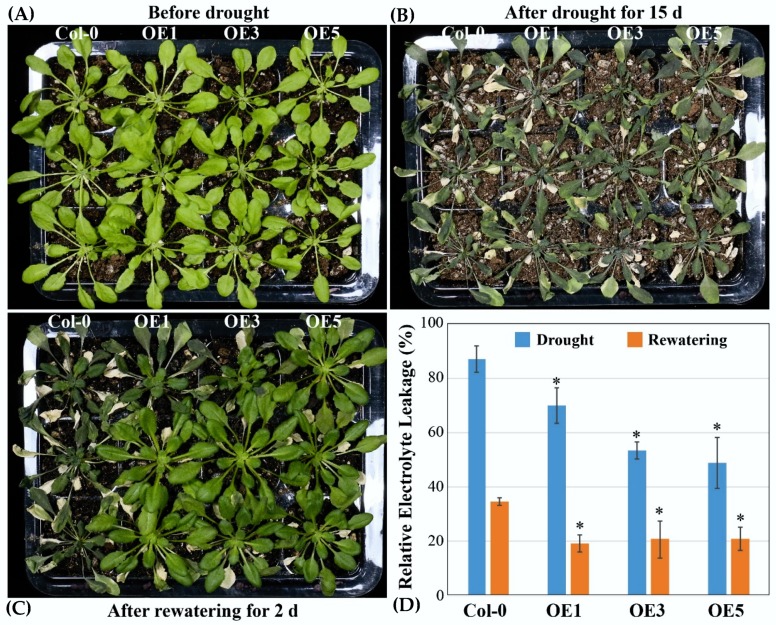
Overexpression of *CsATG18a* enhanced drought tolerance in transgenic *Arabidopsis.* (**A–C**) The representative pictures of three homozygous OE lines (OE1, OE3, and OE5) of *CsATG18a* and WT (Col-0) of *Arabidopsis* that were treated before drought (**A**), after drought for 15 days (**B**), and after rewatering for 2 days (**C**). (**D**) Relative electrolyte leakage of WT and OE lines measured after drought for 15 days and after rewatering for 2 days.

Figure 8 shows that the OE lines of *CsATG18a* showed similar wilting phenotype with WT after 15 days of drought treatment. However, after rewatering for 2 days, all of the OE lines recovered to near normal, whereas the WT still exhibited wilting. In addition, the relative electrolyte leakage of OE lines was significantly lower than that of WT after either drought for 15 days or recovery for 2 days. These results suggest that overexpression of *CsATG18a* conferred drought tolerance in *Arabidopsis*. For the OE lines of *CsATG18b*, we also evaluated their tolerance to drought stress but observed no obvious tolerant phenotype (Figure S1).

### 2.6. Overexpression of CsATG18b Conferred Cold Tolerance in Arabidopsis

**Figure 9:**
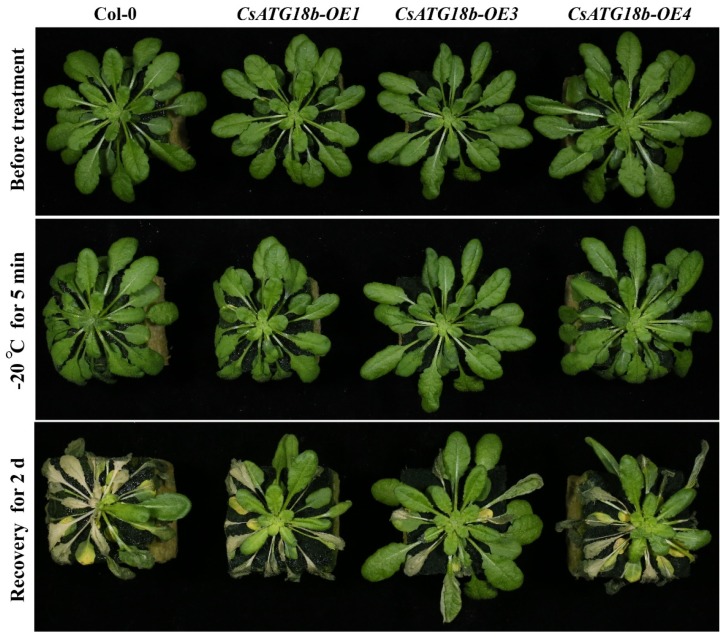
Overexpression of *CsATG18b* reduced leaf damage of transgenic *Arabidopsis* under cold stress. Representative pictures of WT and OE lines (OE1, OE3, and OE4) of *CsATG18b* were photographed before treatment, after −20 °C for 5 min, and after recovery (25 °C) for 2 days.

**Figure 10:**
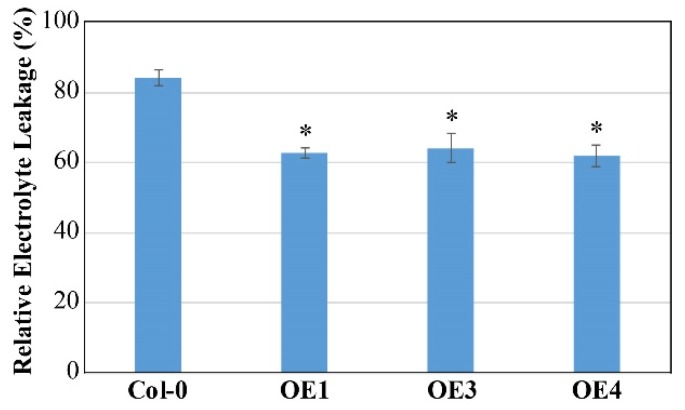
Overexpression of *CsATG18b* decreased relative electrolyte leakage of transgenic *Arabidopsis* under cold stress. Relative electrolyte leakages of WT and OE lines were measured after −20 °C treatment for 5 min. The data are the means ± SE of three independent repeats. * indicates a significant difference at *p* < 0.05 using an LSD test.

As shown in Figure 9, the OE lines of *CsATG18b* had a similar growth phenotype with WT before and after cold treatment (-20 °C). However, after recovery for 2 days, the three OE lines had less leaf damage. In addition, the relative electrolyte leakage of OE lines was significantly lower than that of WT after cold treatment for 5 min (Figure 10). These results suggest that overexpression of *CsATG18b* conferred cold tolerance in *Arabidopsis*. For the OE lines of *CsATG18a*, we did not observe a cold tolerance phenotype (Figure S2).

## 3. Discussion

*ATG* genes are known to be involved in the autophagy process [1]. Most *ATGs* were first identified and functionally characterized by mutagenesis studies in yeast [2]. More than 30 *ATGs* have now been identified in yeast, and 23, including *ATG1-10*, *12-14*, *16-18*, *20*, *27*, *29*, *31*, *TOR*, *VPS15*, and *VPS34*, are considered to be the core *ATGs* participating in autophagy [3,4]. In *Arabidopsis*, around 40 homologues to these core ATGs have been identified, except for *ATG14*, *17*, *27*, *29*, and *31* with no homologues [1,3]. Similarly, a total of 19 core *CsATGs*, including 35 members, were identified from the sweet orange genome in this study. The quantity of *ATG*s in sweet orange is similar to the quantity in grape (35 *VvATGs*) [13], banana (32 *MaATGs*) [12], rice (33 *OsATGs*) [7], and foxtail millet (37 *SiATGs*) [11], suggesting very conservative evolution of ATGs in these plants. Among the 19 core *CsATGs*, we found that *CsATG1*, *CsATG8*, and *CsATG18* contained multiple members, which is similar to that in *Arabidopsis* and other plants [1,8,13]. However, unlike having two *ATG4*, *ATG12*, and *ATG13* genes and one *VTI12* in *Arabidopsis*, sweet orange has one member of *CsATG4*, *CsATG1*2, and *CsATG13* and four members of *CsVTI12*. This indicates a citrus crop-specificity in the number of these *ATGs* [1,8]. On the basis of gene duplication analysis, a high proportion of segmental duplications was predicted for those multiple members of *CsATGs*, especially for *CsATG8*. This suggests that expansion of these *CsATG* subfamilies was possibly derived from gene duplication during evolution [34].

Before functions were identified via transgenic strategy, the potential functions of unknown genes were often predicted according to their orthologous genes, conserved domains, and expression patterns. In this study, 29 of the *CsATG* genes showed higher than 60% sequence identity to homologues of *Arabidopsis*. Most of these high identities of CsATG and AtATG were also closely clustered together in phylogenetic trees. This result indicates that some *CsATG* genes may have functions similar to their homologues in *Arabidopsis*. On the basis of analysis of conserved domains, three *CsATG1* members have similar serine/threonine-protein kinase domains that function in forming a complex with ATG13 and regulating the initiation of autophagy [35]. The UBQ and v-SNARE domains are conserved in all *CsATG8* and *CsVTI12* members, respectively. In general, the similar domain containing in the family members indicates the possible functional redundancy of this family [8]. However, the phylogenetic analysis showed that the members of *CsATG1*, *CsATG8*, and *CsVTI12* were obviously clustered in two different branches, indicating that functional divergences are also existing in these subfamilies. A functional divergence had been reported for *ATG8b* and *ATG8i* during the hydrotropic response in *Arabidopsis* roots [36], which agrees well with their different phylogenetic branches in this study. In the *CsATG18* subfamily, all members contain the WD40 domain, except *CsATG18f*, *CsATG18g*, and *CsATG18h* with an extra BCAS3 domain. The phylogenetic analysis also showed that the *CsATG18f*, *CsATG18g*, and *CsATG18h* were clustered in an independent branch, indicating functional divergence of these three *CsATGs* with the other members in this family [10].

*ATG* genes have played key roles in plant responses to different abiotic stresses. We found that the expression of most *CsATG* genes were induced under drought, heat, cold, mannitol, or salt stresses. Our results support the previous findings regarding the involvement of autophagy in abiotic stresses [3,25,28]. Although most *CsATGs* were induced by these stresses, some *CsATGs* showed different expression patterns to different stresses. For example, *CsATG1a* was significantly up-regulated under drought and heat but down-regulated under cold. In contrast, *CsATG1d* was significantly up-regulated under cold. In the *CsATG8* subfamily, *CsATG8a*, *CsATG8c*, *CsATG8g*, and *CsATG8i* were obviously induced by drought and heat, but this was not true for *CsATG8d* and *CsATG8f.* Similar results have been reported for *ATGs* of other plant species, such as *VvATGs* of grape [13], *NtATGs* of tobacco [8], and *CaATGs* of pepper [10]. These results indicate that there may be different pathways to regulate the autophagy process under different abiotic stresses [10,15,25]. Compared to the above tested abiotic stresses, the roles of autophagy in heavy metals toxicity are less well known in plants. To provide more evidence, expression patterns of *CsATGs* were analyzed under high levels of Cu, Mn, and Cd. Unlike the predominant up-regulation of *CsATGs* under drought, heat, cold, and salt stress, more down-regulated *CsATGs* were found upon exposure to excess Cu, Mn, and Cd. In addition, the expression patterns were different between heavy metal-treated roots and leaves. Although we could not find similar reports to support our result at present, these data provide a new reference for understanding potential roles of ATGs in heavy metal stress.

ATG18 is a WD40 domain containing protein family with multiple members. They can function in vesicle formation in autophagy by binding to the phosphatidylinositol 3-phosphate sites in plants [2]. Among the multiple ATG18 members, ATG18a is the most studied. *RNAi-AtATG18a* transgenic *Arabidopsis* plants were autophagy-defective and more sensitive to oxidative, salt, and drought stresses than WT [25,37]. *MdATG18a* of apple showed significant up-regulation in response to leaf senescence, drought, heat, oxidative stress, and nitrogen starvation, and overexpression of *MdATG18a* in tomato and apple enhanced drought tolerance [21,38]. In the present study, overexpression of a *CsATG18a* in *Arabidopsis* also improved tolerance to osmotic stress, salt, and drought, which is consistent with previous results. Another member, *CsATG18b*, was shown to confer enhanced tolerance to osmotic stress, salt, and cold. This result provides new evidence regarding the functions of *ATG18b* in plants. Under abiotic stresses, a common response in cells is ROS accumulation and oxidative damage, including membrane breakage and protein oxidation and aggregation [21,37]. Thus, we speculate that enhanced tolerance in *CsATG18a* and *CsATG18b* transgenic plants might be because the damaged proteins and components are more efficiently degraded and recycled by the autophagy pathway, thereby reducing ROS accumulation and maintaining membrane integrity under stress [37,39]. Interestingly, a functional difference between *CsATG18a* and *CsATG18b* was noticed under drought and cold. Overexpression of *CsATG18a* conferred drought but not cold tolerance, whereas overexpression of *CsATG18b* conferred cold but not drought tolerance. This result appears reasonable and explainable. First, *CsATG18a* and *CsATG18b* have very different gene structures and predicted subcellular localization, which indicates their functional divergence (Table 1 and Figure 3). Second, qRT-PCR analysis shows that *CsATG18a* has a higher expression level under drought but a lower expression level under cold than *CsATG18b* (Figure 4). Third, *CsATG18a* and *CsATG18b* may be regulated by different transcription factors (TFs) under drought and cold. There is evidence that *ATG18a* interacts with the WRKY33 transcription factor to mediate autophagosome formation and necrotrophic pathogen resistance in *Arabidopsis* [40]. The *HsfA1a* transcription factor binds to the *ATG18f* promoter to regulate drought tolerance in tomato [27]. *ATG18a* and *ATG18f* are regulated by two different TFs, indicating that *CsATG18a* and *CsATG18b* may be also regulated by different TFs, thereby resulting in their functional differentiations. 

## 4. Materials and Methods

### 4.1. Plant Materials and Treatments

“Hamlin” sweet orange (*Citrus sinensis* (L.) Osbeck) was used as the plant material in this study. The seeds of “Hamlin” sweet orange were collected from the Citrus Research Institute, Southwest University, Chongqing, China. The seeds were sterilized and germinated, and the seedlings were hydroponically cultured at 25 °C and 16 h photoperiod (50 µmol m^−2^ s^−1^), as described by Fu et al. [41]. After 30 days of growth in hydroponic solution, uniform seedlings were divided into different groups (3 biological repeats and 10 plants per repeat for each group) for the following sets of treatments. For the drought treatment, the seedlings were dehydrated under room temperature (25°C) by removing the hydroponic solution. For the heat and cold treatments, two groups of seedlings were put in a growth chamber under 42 °C and a refrigerator under 4 °C, respectively. For the mannitol and NaCl treatments, two groups of seedlings were transferred to 400 mM mannitol and 200 mM NaCl solutions, respectively. For the excess Cu, Mn, and Cd treatments, the seedlings were transferred to new hydroponic solutions containing 0.01 mM CuSO4 (+Cu), 0.1 mM MnSO4 (+Mn), and 0.038 mM CdSO4 (+Cd), respectively. The seedlings grown under normal conditions were used as controls (CK). The leaves were sampled after 3 h and 12 h of drought, heat, and cold treatments or 2 h and 3 h of mannitol and NaCl treatments, and the leaves and roots were sampled upon 5 days and 15 days of excess Cu, Mn, and Cd treatments. Samples were immediately frozen in liquid nitrogen and stored at −80 °C until use.

### 4.2. Genome-Wide Identification of ATG Genes in Sweet Orange

To identify all putative ATG proteins of the sweet orange, a BLASTP search of the sweet orange genome database (http://citrus.hzau.edu.cn/orange/) [34] was performed using the known *Arabidopsis* ATG proteins (AtATGs) as query sequences [1,3]. The results were filtered using a score value of ≥ 100 and an *e*-value ≤ e^−10^ [42]. After removal of redundant sequences, all putative *ATG* genes were submitted to the PFAM database (http://pfam.xfam.org/) to confirm the existence of the ATG domains. All of the identified *ATG* genes of sweet orange were named as *CsATGs*.

### 4.3. Bioinformatic Analysis of CsATGs

The subcellular locations of the CsATG proteins were predicted online by using the ProtComp v. 9.0 (http://www.softberry.com/berry.phtml?topic=protcomppl&group=programs&subgroup=proloc). To construct phylogenetic trees, all putative CsATGs and the ATG proteins from *Arabidopsis thaliana* [1,3], tobacco (*Nicotiana tabacum*) [8], rice (*Oryza sativa*) [7], and grapevine (*Vitis vinifera*) [13] were first collected (as shown in Table S1) and then submitted to ClustalW for performing multiple sequence alignment [43]. The generated files were used to construct the phylogenetic tree with the neighbor-joining method and 1000 replicates of bootstrap analysis in MEGA 6 software [44]. The bootstrap values lower than 50% are not shown in the phylogenetic trees. The chromosomal positions of the *CsATG* genes were provided by the sweet orange genome database, and MapInspect software (http://mapinspect.software.informer.com) was used to draw the location images. For the gene structure analysis, the generic feature format (GFF) file of each CsATG was downloaded from the sweet orange genome database and then submitted to the Gene Structure Display Server (GSDS, http://gsds.cbi.pku.edu.cn/) to display intron-exon structures [45]. The GFF files were also inputted into MCScanX software to identify gene duplications and collinear correlations [46]. The conserved domains of CsATG proteins were predicted online in the NCBI Conserved Domain Database (https://www.ncbi.nlm.nih.gov/cdd/).

### 4.4. Total RNA Isolation and Quantitative Real-Time PCR (qRT-PCR) Analysis

Total RNA was extracted from the CK and stress-treated leaves or roots of “Hamlin” sweet orange seedlings using a RNAprep pure plant kit (Tiangen Biotech Co., Ltd., Beijing, China), and the RNA concentration and quality were determined with a Nanodrop 2000 spectrophotometer (Thermo Scientific, Waltham, MA, USA). Then, 1 μg of high-quality RNA was used for cDNA synthesis with an iScript cDNA synthesis kit (Bio-Rad) according to the manufacturer’s instructions. qRT-PCR was performed on the Bio-Rad CFX Connect RealTime system using ChamQ Universal SYBR qPCR Master Mix (Vazyme Biotech Co., Ltd., Nanjing, China). Each PCR reaction contained 5.0 μL SYBR mix, 0.2 μM primers, and 1.0 μL diluted cDNA in a final volume of 10 μL. Specific primers of *CsATG* genes were designed using the online primer-blast program in the NCBI website, whereas *Actin* (Cs1g05000.1) was used as a reference gene to normalize the relative expression levels of the tested genes. Three biological replicates and three technical replicates were performed for each treatment.

### 4.5. Construction of Transgenic Arabidopsis Expressing 35S-CsATG18a and 35S-CsATG18b

The CDS (coding sequence) fragments of *CsATG18a* and *CsATG18b* were first isolated from “Hamlin” sweet orange. After being confirmed by sequencing, the full CDS fragments of *CsATG18a* and *CsATG18b* were recombined into a pGBO vector to produce *35S-CsATG18a* and *35S-CsATG18b* constructs, respectively. The two constructs were then transformed into *Agrobacterium tumefaciens* strain GV3101 by electroporation. The *Arabidopsis thaliana* wild-type (WT; ecotype Col-0) plants were used to perform *Agrobacterium*-mediated transformation by the floral dipping method [47]. The transgenic plants were selected on 1/2 MS medium containing 50 mg L^−1^ kanamycin until a homozygous T4 generation was obtained. The expression levels of transgenes were measured by qRT-PCR.

### 4.6. Phenotypic and Physiological Analysis of Transgenic Arabidopsis Lines

To evaluate the tolerance of *CsATG18a* and *CsATG18b* transgenic *Arabidopsis* plants to different abiotic stresses, three independent T4 homozygous lines with relatively high expression levels of *CsATG18a* and *CsATG18b* were selected for NaCl, mannitol, drought, and cold treatments, and WT (Col-0) plants were used as controls. For the NaCl and mannitol treatments, the seeds of transgenic lines and WT were sown on 1/2 MS with 200 mM NaCl or 400 mM mannitol, and the germination rate was calculated at 3 to 7 days. For the drought treatment, 30 day old transgenic and WT plants were dewatered for 15 days and then watered for recovery. For the cold treatment, 30 day old transgenic and WT plants were transferred to a −20 °C freezer for 5 min and then back to room temperature for recovery. After drought and cold treatments, the relative electrolytic leakage was measured as described by Shi et al. [48].

## 5. Conclusions

A total of 35 *CsATG* genes were identified from the genome of sweet orange. These CsATGs showed high sequence and phylogeny similarities with the AtATGs of *Arabidopsis*. Most CsATG genes were significantly up- or down-regulated in response to drought, heat, cold, salt, and osmotic stresses, as well as to excess Cu, Mn, and Cd stresses. Overexpression of *CsATG18a* and *CsATG18b* enhanced tolerance to both salt and osmotic stresses in *Arabidopsis*. Overexpression of *CsATG18a* in *Arabidopsis* conferred drought but not cold tolerance, whereas overexpression of *CsATG18b* conferred cold but not drought tolerance. This study analyzed *CsATG* genes in citrus and preliminarily revealed their important roles in response to abiotic stresses. In future, their functions and the underlying mechanisms will be further uncovered by overexpressing in citrus plants.

## Figures and Tables

**Table 1:** Detailed information of autophagy-related genes (ATGs) in *Arabidopsis* and sweet orange.

Gene	*Arabidopsis* ID	Gene	Citrus ID	Identity to *Arabidopsis*	ORF (bp)	Protein (aa)	Predicted Localization ^1^	Predicted Function ^2^
**ATG1/13 kinase complex**								**Initiation of autophagy**
*AtATG1a*	At1g49180	***CsATG1a***	Cs1g05170	56.3%	894	297	Cytoplasmic	Serine/threonine-protein kinase
*AtATG1b*	At2g37840	***CsATG1b***	orange1.1t03071	66.5%	2157	718	Nuclear	Serine/threonine-protein kinase
*AtATG1c*	At3g53930	NA	NA	NA	NA	NA	NA	NA
*AtATG1d*	At3g61960	***CsATG1d***	orange1.1t00581	59.1%	2079	692	Cytoplasmic	Serine/threonine-protein kinase
*AtATG13a*	At3g18770	***CsATG13***	Cs1g05100	61.7%	3222	1073	Cytoplasmic	Autophagocytosis-associated protein
*AtATG13b*	AT3G49590	NA	NA	NA	NA	NA	NA	NA
*AtATG20*	At5g06140	***CsATG20***	Cs6g14260	83.3%	1212	403	Mitochondrial	Sorting nexin 2-like protein
*AtTOR*	At1g50030	***CsTOR***	Cs8g03630	83.6%	7095	2364	Cytoplasmic	Phosphoinositide 3-kinase
**PI3K complex**								**Autophagosome formation**
*AtVPS15*	At4g29380	***CsVPS15***	Cs4g15580	73.7%	4662	1553	Cytoplasmic	Serine/threonine-protein kinase
*AtVPS34*	At1g60490	***CsVPS34***	Cs7g08800	83.8%	2469	822	Cytoplasmic	Phosphoinositide 3-kinase
*AtATG6*	At3g61710	***CsATG6***	orange1.1t00487	77.4%	1551	516	Cytoplasmic	Autophagy protein Apg6
**ATG9/2/18 complex**								**Membrane recruitment to autophagosome**
*AtATG2*	At3g19190	***CsATG2***	Cs8g05820	51.6%	5985	1994	Plasma membrane	Autophagy-related protein 2
*AtATG9*	At2g31260	***CsATG9***	Cs5g29970	69.9%	2625	874	Vacuolar	Autophagy protein Apg9
*AtATG18a*	At3g62770	***CsATG18a***	Cs5g32770	76.5%	1221	406	Cytoplasmic	WD-40 repeat containing protein
*AtATG18b*	At4g30510	***CsATG18b***	Cs4g02320	74.2%	1095	364	Mitochondrial	WD-40 repeat containing protein
*AtATG18c*	At2g40810	***CsATG18c***	Cs7g29360	75.6%	1248	415	Mitochondrial	WD-40 repeat containing protein
*AtATG18d*	At3g56440	NA	NA	NA	NA	NA	NA	NA
*AtATG18e*	At5g05150	***CsATG18e***	Cs3g14170	47.6%	1083	360	Cytoplasmic	WD-40 repeat containing protein
*AtATG18f*	At5g54730	***CsATG18f***	Cs4g11290	48.7%	2340	779	Plasma membrane	Breast carcinoma amplified sequence 3
*AtATG18g*	At1g03380	***CsATG18g***	Cs5g32120	57.5%	2985	994	Plasma membrane	Breast carcinoma amplified sequence 3
*AtATG18h*	At1g54710	***CsATG18h***	Cs5g34870	62.0%	3021	1006	Mitochondrial	Breast carcinoma amplified sequence 3
**Ubiquitin-like ATG8 and PE conjugation pathway**								**Conjugation of ATG8 to PE**
*AtATG3*	At5g61500	***CsATG3***	Cs9g15060	82.1%	960	319	Cytoplasmic	Autophagocytosis-associated protein 3
*AtATG4a*	At2g44140	NA	NA	NA	NA	NA	NA	NA
*AtATG4b*	At3g59950	***CsATG4***	Cs5g20640	60.8%	1332	443	Cytoplasmic	Peptidase family C54
*AtATG7*	At5g45900	***CsATG7***	Cs2g04910	71.9%	2148	715	Cytoplasmic	Autophagy protein Apg7
*AtATG8a*	At4g21980	***CsATG8a***	Cs2g03030	88.2%	363	120	Cytoplasmic	Autophagy protein Apg8
*AtATG8b*	AT4G04620	NA	NA	NA	NA	NA	NA	NA
*AtATG8c*	At1g62040	***CsATG8c***	Cs4g10210	90.8%	363	120	Cytoplasmic	Autophagy protein Apg8
*AtATG8d*	At2g05630	***CsATG8d***	Cs2g21350	90.8%	360	119	Mitochondrial	Autophagy protein Apg8
*AtATG8f*	At4g16520	***CsATG8f***	Cs5g08990	90.9%	369	122	Cytoplasmic	Autophagy protein Apg8
*AtATG8g*	At3g60640	***CsATG8g***	orange1.1t00201	84.6%	354	117	Mitochondrial	Autophagy protein Apg8
*AtATG8h*	At3g06420	NA	NA	NA	NA	NA	NA	NA
*AtATG8i*	At3g15580	***CsATG8i***	orange1.1t01753	79.1%	378	125	Mitochondrial	Autophagy protein Apg8
**Ubiquitin-like ATG12 and ATG5 conjugation pathway**								**Conjugation of ATG12, ATG5, and ATG16**
*AtATG5*	At5g17290	***CsATG5***	Cs9g11540	70.2%	1233	410	Cytoplasmic	Autophagy protein Apg5
*AtATG7*	At5g45900	***CsATG7***	Cs2g04910	71.9%	2148	715	Cytoplasmic	Autophagy protein Apg7
*AtATG10*	At3g07525	***CsATG10***	Cs2g18190	56.1%	693	230	Plasma membrane	Autophagocytosis-associated protein
*AtATG12a*	At1g54210	***CsATG12***	Cs1g17600	81.1%	315	104	Cytoplasmic	Autophagocytosis-associated protein
*AtATG12b*	At3g13970	NA	NA	NA	NA	NA	NA	NA
*AtATG16*	At5g50230	***CsATG16***	Cs5g06560	74.1%	1533	510	Cytoplasmic	WD-40 repeat containing protein
**SNARE**								**Fusion of autophagososme with the vacuole**
*AtVTI12*	At1g26670	***CsVTI12a***	orange1.1t04711	70.1%	666	221	Golgi	Vesicle transport v-SNARE protein
		***CsVTI12b***	Cs7g03410	72.7%	519	172	Golgi	Vesicle transport v-SNARE protein
		***CsVTI12c***	Cs6g21610	68.3%	666	221	Golgi	Vesicle transport v-SNARE protein
		***CsVTI12d***	orange1.1t00788	71.7%	522	173	Golgi	Vesicle transport v-SNARE protein

^1^ Localizations of CsATGs (*Citrus sinensis*, Cs) were prediceted online by using the ProtComp. ^2^ Functions of CsATGs were predicted according to the annotations of AtATGs (*Arabidopsis thaliana*, At) in the *Arabidopsis* information resource (TAIR) database.

## Supplementary Materials

Supplementary materials can be found at https://www.mdpi.com/1422-0067/21/8/2699/s1.

## Abbreviations

TOR	target of rapamycin kinase
PE	phosphatidylethanolamine
ROS	reactive oxygen species
PCD	programmed cell death
UTR	untranslated region
FC	fold change
OE	overexpression
PKc like	protein kinase superfamily
ATG C	autophagy-related protein C-terminal domain
ATG N	autophagy-related protein N terminal domain
Chorein N	N-terminal region of chorein or VPS13
Autophagy N	autophagocytosis-associated protein N-terminal domain
Autophagy C	autophagocytosis-associated protein C-terminal domain
Autophagy act C	autophagocytosis-associated protein, active-site domain
Peptidase C54	peptidase family C54
UBQ	ubiquitin homologues
WD40 Repeats	WD40 repeats
BCAS3	breast carcinoma amplified sequence 3
PX domain	the phox homology domain, a phosphoinositide binding module
BAR	the Bin/amphiphysin/Rvs (BAR) domain
PI3Kc	catalytic domain of phosphoinositide 3-kinase
PI3Ka	phosphoinositide 3-kinase family, accessory domain
C2	C2 domain
SNARE	soluble *N*-ethylmaleimide-sensitive-factor attachment protein receptor
V-SNARE N	vesicle transport v-SNARE protein N-terminus
V-SNARE C	vesicle transport v-SNARE protein C-terminus
