# Supplementary material for: Comprehensive Analysis of Autophagy-Related Genes in Sweet Orange (Citrus sinensis) Highlights Their Roles in Response to Abiotic Stresses
Source: Int J Mol Sci. 2020 Apr 13;21(8):2699. doi: 10.3390/ijms21082699 (PMC7215763; doi:10.3390/ijms21082699)
Supplement: Supplementary file 1 [file ijms-21-02699-s001.zip › supplementary figures]

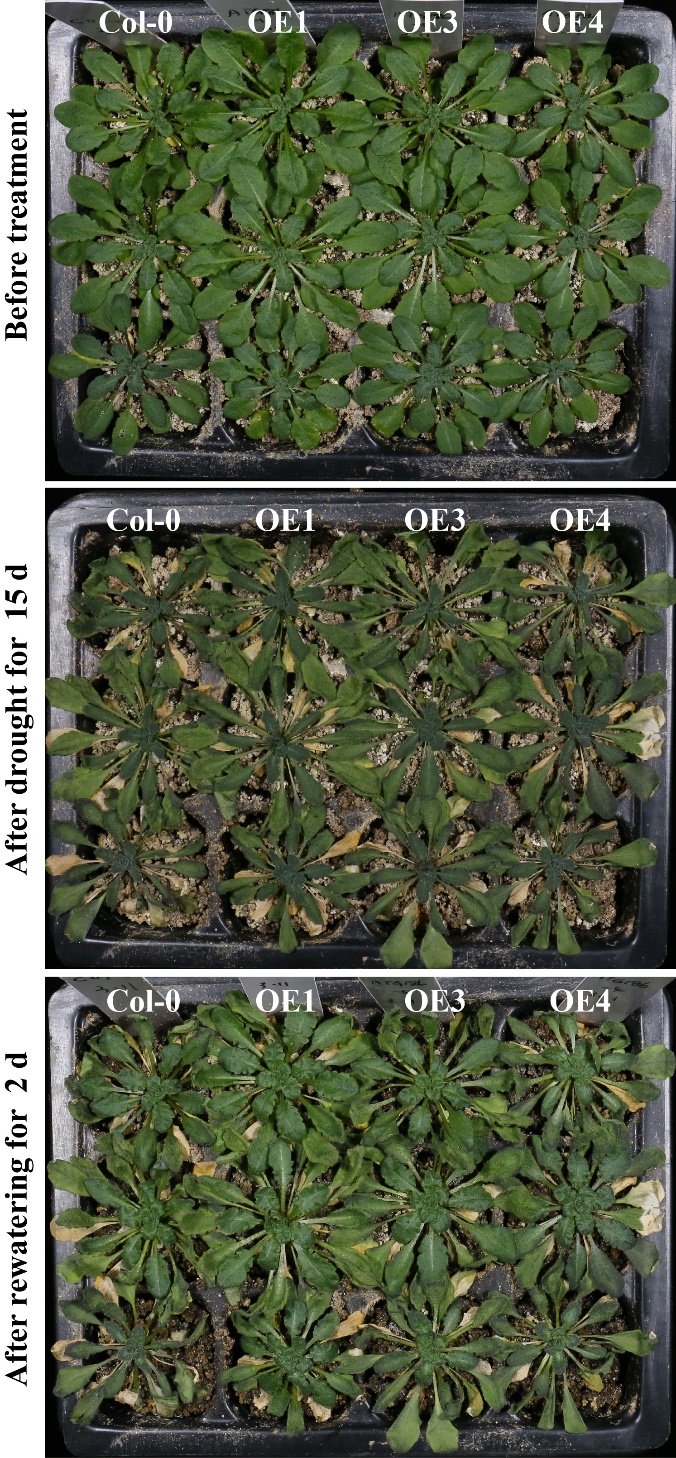


**Figure S1.** The representative pictures of three homozygous OE lines (OE1, OE3, and OE4) of *CsATG18b* and WT (Col-0) of *Arabidopsis* that were treated before drought, after drought for 15 d, and after rewatering for 2 d. No obvious tolerant phenotype was observed in *CsATG18b* overexpressed *Arabidopsis* under drought.
